# Supplementary material for: Tissue-specific clonal selection and differentiation of CD4+ T cells during infection
Source: Nat Immunol. Author manuscript; Available in PMC 2026 Aug 21. (PMC13498420; doi:10.1038/s41590-026-02451-4)
Supplement: Supplementary file 1 [file NIHMS2201428-supplement-1.pdf]

A

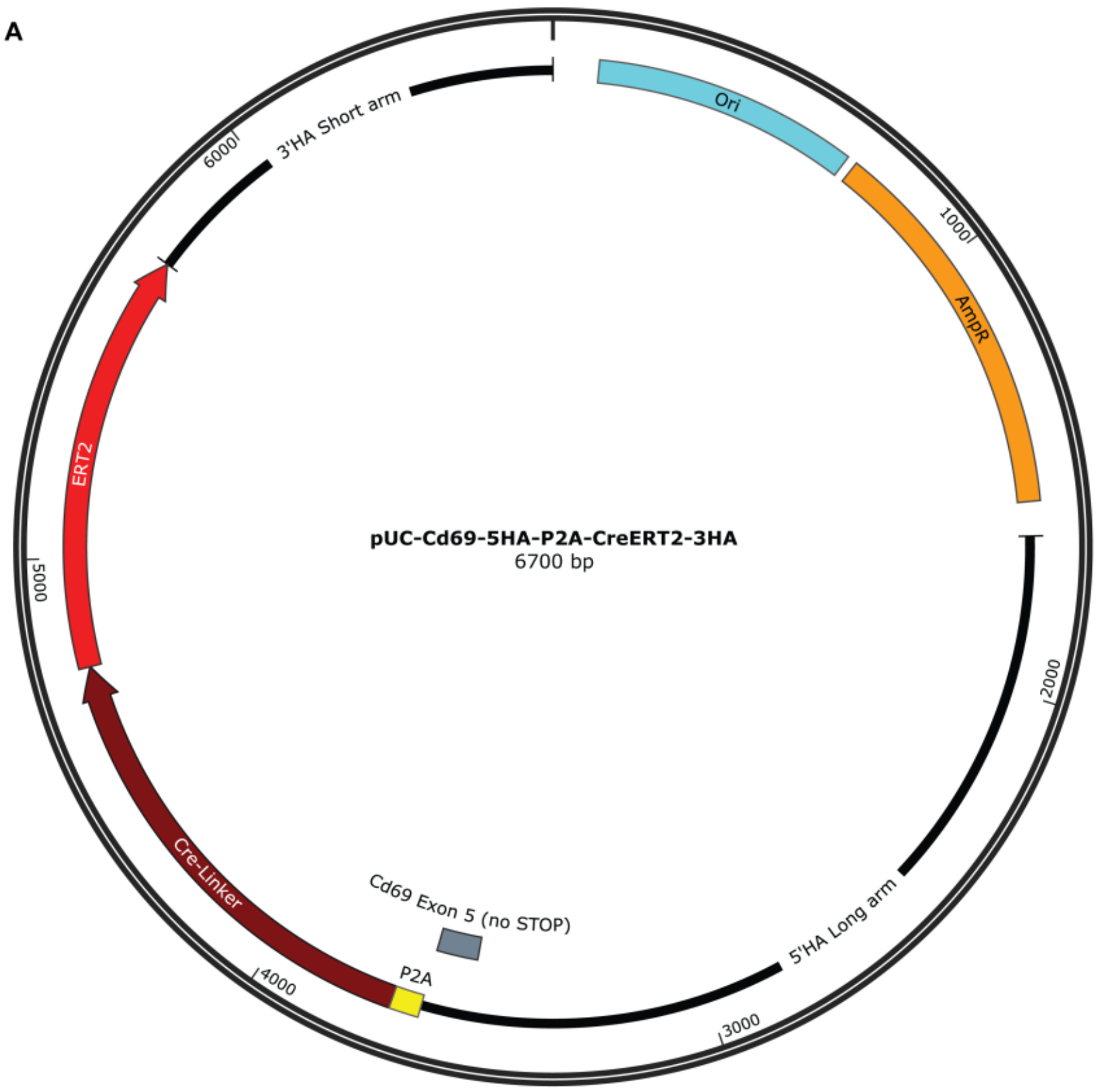

B

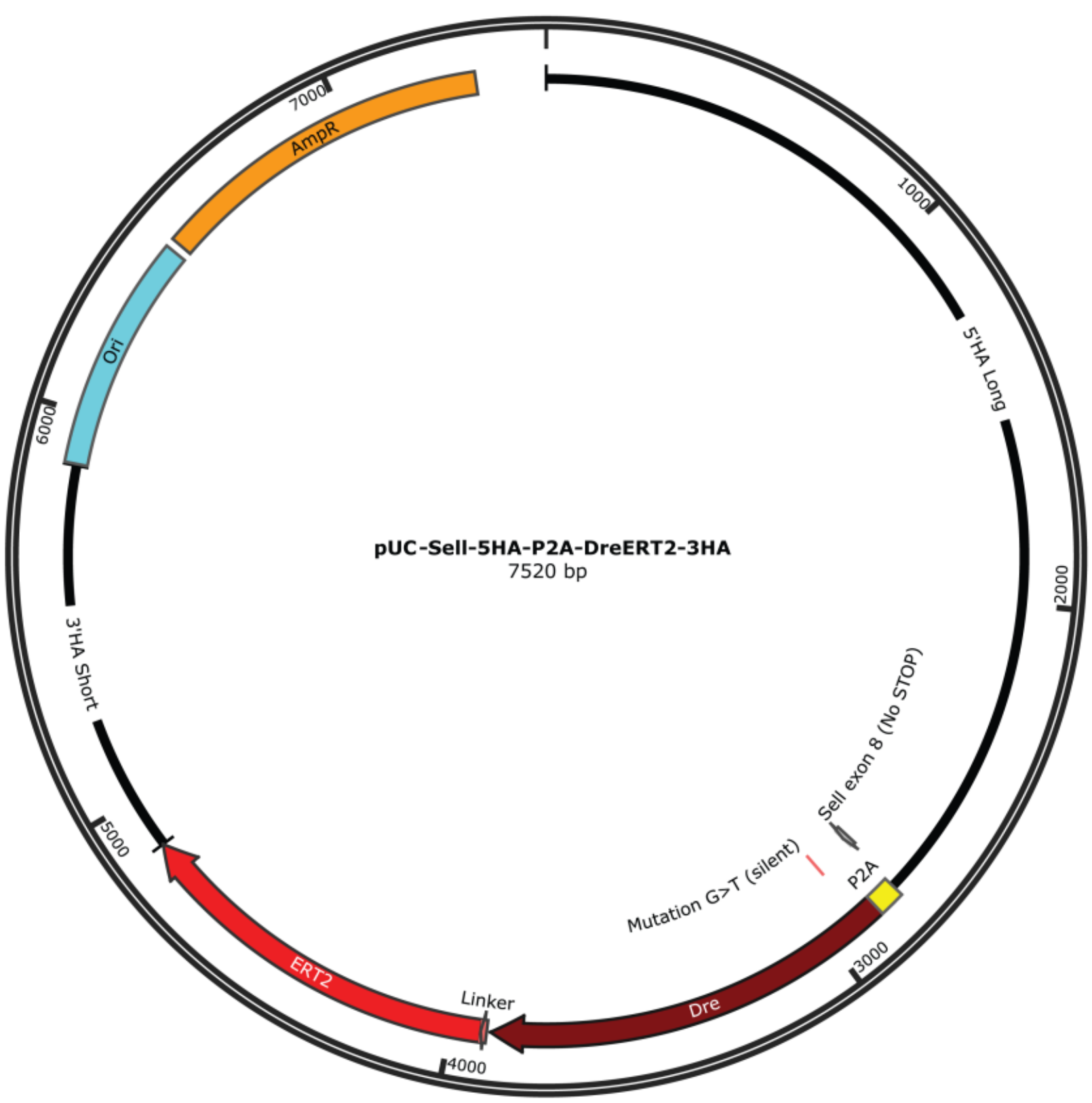

Supplementary Fig. 1: Target vector plasmids for Cd69-CreERT² and Sell-DreERT² mice.
(**A**) Cd69-CreERT2 plasmid (**B**) Sell-DreERT2 plasmid

TCR ID	Clone	CDR3 A	CDR3 B	Va-gene	Ja-gene	Vb-gene	Db-gene	Jb-gene
RP_TCR1	TRA_TRB_14.1_A9_Lung1	CAADDTNAYKVIF	CTCSGDWPYEQYF	Trav4-4-dv10	Traj30	Trbv1	Trbd2	Trbj2-7
RP_TCR2	TRA_TRB_24.1_A9_Lung2	CAASRTNSAGNKLTF	CAWSPEYEQYF	Trav7d-2	Traj17	Trbv31	Trbd1	Trbj2-7
RP_TCR3	TRA_TRB_29.1_A9_Lung3	CAASSWSNYQLIW	CAWKQRRSAETLYF	Trav7d-2	Traj33	Trbv31	Trbd2	Trbj2-3
RP_TCR4	TRA_TRB_19.1_A9_Lung4	CAASPNYNVLYF	CTCSADINSDYTF	Trav14-1	Traj21	Trbv1		Trbj1-2
RP_TCR5	TRA_TRB_12.1_A9_Lung5	CAARGSNYQLIW	CASSPGTVSNERLFF	Trav14-2	Traj33	Trbv26	Trbd1	Trbj1-4
RP_TCR6	TRA_TRB_34.1_A9_medLN1	CAASSNYNVLYF	CAWTGTGGAYAEQFF	Trav7d-2	Traj21	Trbv31	Trbd2	Trbj2-1
RP_TCR7	TRA_TRB_9.1_A9_medLN2	CAMRDLGNNNAPRF	CASSDGQGYQAPLF	Trav6-3	Traj43	Trbv13-3	Trbd1	Trbj1-5
RP_TCR8	TRA_TRB_32.2_A9_medLN3	CAARASSGSWQLIF	CASSQDWGGAFEQYF	Trav7-4	Traj22	Trbv26	Trbd2	Trbj2-7
RP_TCR9	TRA_TRB_13.2_A9_medLN4	CALSDQTGFASALTF	CASSSGGNTEVFF	Trav6-7	Traj35	Trbv4	Trbd2	Trbj1-1
RP_TCR10	TRA_TRB_72.1_A9_medLN5	CAVSIITGNTGKLIF	CASSLEGTQYF	Trav3n-3	Traj37	Trbv3	Trbd1	Trbj2-5
RP_TCR11	TRA_TRB_4.3_A9_Spleen1	CAVSAGSNYNVLYF	CASSLGWASQNTLYF	Trav9-1	Traj21	Trbv16	Trbd2	Trbj2-4
RP_TCR12	TRA_TRB_7.1_A9_Spleen2	CAVSAMSNYNVLYF	CASSIPSDRGHEQYF	Trav9-1	Traj21	Trbv19	Trbd1	Trbj2-7
RP_TCR5	TRA_TRB_12.1_A9_Spleen3	CAARGSNYQLIW	CASSPGTVSNERLFF	Trav14-2	Traj33	Trbv26	Trbd1	Trbj1-4
RP_TCR13	TRA_TRB_27.1_A9_Spleen4	CALTPSSSSFSKLVF	CASSRGQGNTEVFF	Trav6-5	Traj50	Trbv2	Trbd1	Trbj1-1
RP_TCR14	TRA_TRB_1.10_A9_Spleen5	CALSDPNQGGSAKLIF	CASSQTGGAREQYF	Trav6-7-dv9	Traj57	Trbv3	Trbd2	Trbj2-7
RP_TCR15	TRA_TRB_50.1_A9_Spleen6	CAIDPYNTNTGKLTF	CAWSLEYEQYF	Trav13n-2	Traj27	Trbv31		Trbj2-7
RP_TCR16	NP_A9_TP_Lung1	CALSDRNQGGSAKLIF	CASSLSVGGEQYF	Trav6-7-dv9	Traj57	Trbv3	Trbd2	Trbj2-7
RP_TCR17	NP_A9_TN_Lung2	CILRALNQGGSAKLIF	CASSQQTSAETLYF	Trav21	Traj57	Trbv5	Trbd2	Trbj2-3
RP_TCR18	NP_A9_TN_Lung3	CALSVPNQGGSAKLIF	CASSQTGGAREQYF	Trav6-7-dv9	Traj57	Trbv3	Trbd2	Trbj2-7
RP_TCR19	NP_A9_TP_Lung4	CALSDPNQGGSAKLIF	CASSRTGGAREQYF	Trav6-7-dv9	Traj57	Trbv3	Trbd2	Trbj2-7
RP_TCR20	NP_A9_NP_Lung5	CVLGTFNSGGSNAKLTF	CASSQTSAETLYF	Trav6-2	Traj42	Trbv2	Trbd2	Trbj2-3
RP_TCR19	NP_A9_TP_medLN1	CALSDPNQGGSAKLIF	CASSRTGGAREQYF	Trav6-7-dv9	Traj57	Trbv3	Trbd2	Trbj2-7
RP_TCR21	NP_A9_TP_medLN2	CAASASSGSWQLIF	CASSRTGDTEVFF	Trav14n-2	Traj22	Trbv2	Trbd1	Trbj1-1
RP_TCR16	NP_A9_TP_medLN3	CALSDRNQGGSAKLIF	CASSLSVGGEQYF	Trav6-7-dv9	Traj57	Trbv3	Trbd2	Trbj2-7
RP_TCR17	NP_A9_TN_medLN4	CILRALNQGGSAKLIF	CASSQQTSAETLYF	Trav21	Traj57	Trbv5	Trbd2	Trbj2-3
RP_TCR18	NP_A9_TN_medLN5	CALSVPNQGGSAKLIF	CASSQTGGAREQYF	Trav6-7-dv9	Traj57	Trbv3	Trbd2	Trbj2-7
RP_TCR22	NP_A9_TP_medLN6	CAASANTGNYKYVF	CASSLQYEQYF	Trav14-1	Traj40	Trbv3	Trbd1	Trbj2-7
RP_TCR20	NP_A9_TP_medLN7	CVLGTFNSGGSNAKLTF	CASSQTSAETLYF	Trav6-2	Traj42	Trbv2	Trbd2	Trbj2-3
RP_TCR23	NP_A9_TN_medLN8	CAMREAGNTGKLIF	CASSSDRGNSDYTF	Trav16	Traj37	Trbv3	Trbd1	Trbj1-2
RP_TCR24	NP_A9_TP_medLN9	CAMRGGGNTGKLIF	CASSSDRPNSDYTF	Trav16	Traj37	Trbv3	Trbd1	Trbj1-2
RP_TCR25	NP_A9_TN_medLN10	CVLSAASSFSKLVF	CASSLGISNERLFF	Trav9-2	Traj50	Trbv3	Trbd1	Trbj1-4
RP_TCR19	NP_A9_TP_Spleen1	CALSDPNQGGSAKLIF	CASSRTGGAREQYF	Trav6-7-dv9	Traj57	Trbv3	Trbd2	Trbj2-7
RP_TCR16	NP_A9_TP_Spleen2	CALSDRNQGGSAKLIF	CASSLSVGGEQYF	Trav6-7-dv9	Traj57	Trbv3	Trbd2	Trbj2-7
RP_TCR26	NP_A9_TP_Spleen3	CAVIGSNTNKVVF	CTCSELGGRYAEQFF	Trav9-1	Traj34	Trbv1	Trbd2	Trbj2-1
RP_TCR27	NP_A9_TN_Spleen4	CALSASNQGGSAKLIF	CASSLTGGAREQYF	Trav6-7-dv9	Traj57	Trbv3	Trbd2	Trbj2-7
RP_TCR28	NP_A9_TN_Spleen5	CAMRENSGTYQRF	CASSPGLNNQAPLF	Trav16	Traj13	Trbv3	Trbd1	Trbj1-5
RP_TCR29	NP_A9_TP_Spleen6	CALAPASSGSWQLIF	CASSPTSSGNTLYF	Trav6d-4	Traj22	Trbv5	Trbd1	Trbj1-3
RP_TCR30	NP_A9_TP_Spleen7	CAMRSGNTGKLIF	CASSRDNPNSDYTF	Trav16	Traj37	Trbv13-1	Trbd1	Trbv13-3

RP_TCR31	TRA_TRB_2.2_B56_Lung1	CILRVPATGGNNKLTF	CASSFNQDTQYF	Trav21-dv12	Traj56	Trbv10		Trbj2-5
RP_TCR32	TRA_TRA_TRB_1.1_B56_Lung2	CAADNMGYKLTF	CASGGRDRGREQYF	Trav7d-2	Traj9	Trbv13-2	Trbd1	Trbj2-7
RP_TCR33	TRA_TRB_2.4_B56_Lung3	CILRVPATGGNNKLTF	CASSFNQDTQYF	Trav21-dv12	Traj56	Trbv10		Trbj2-5
RP_TCR34	TRA_TRB_19.1_B56_Lung4	CAVSMSNYNVLYF	CASSQDQGNAEQFF	Trav7d-3	Traj21	Trbv5		Trbj2-1
RP_TCR35	TRA_TRB_3.1_B56_Lung5	CAASFYQGGRALIF	CTCSADTGAGNTLYF	Trav7d-4	Traj15	Trbv1	Trbd1	Trbj1-3
RP_TCR35	TRA_TRB_3.1_B56_medLN1	CAASFYQGGRALIF	CTCSADTGAGNTLYF	Trav7d-4	Traj15	Trbv1	Trbd1	Trbj1-3
RP_TCR36	TRA_TRB_1.1_B56_medLN2	CALSDQNQGGSAKLIF	CASSLTGGAREQYF	Trav6-7-dv9	Traj57	Trbv3		Trbj2-7
RP_TCR37	TRA_TRB_1.3_B56_medLN3	CALSDSNQGGSAKLIF	CASSRTGGAREQYF	Trav6-7-dv9	Traj57	Trbv3		Trbj2-7
RP_TCR38	TRA_TRB_12.1_B56_medLN4	CAMSRATGGNNKLTF	CASSPGTGNSDYTF	Trav16	Traj56	Trbv16	Trbd1	Trbj1-2
RP_TCR33	TRA_TRB_2.4_B56_medLN5	CILRVPATGGNNKLTF	CASSFNQDTQYF	Trav21-dv12	Traj56	Trbv10		Trbj2-5
RP_TCR31	TRA_TRB_2.2_B56_medLN6	CILRVPATGGNNKLTF	CASSFNQDTQYF	Trav21-dv12	Traj56	Trbv10		Trbj2-5
RP_TCR39	TRA_TRB_1.2_B56_medLN7	CALSDPNQGGSAKLIF	CASSLTGGAREQYF	Trav6-7-dv9	Traj57	Trbv3		Trbj2-7
RP_TCR40	TRA_TRB_24.1_B56_medLN8	CAIEGNNRIFF	CTCSVSGSSNERLFF	Trav13d-1	Traj31	Trbv1		Trbj1-4
RP_TCR31	TRA_TRB_2.2_B56_Spleen1	CILRVPATGGNNKLTF	CASSFNQDTQYF	Trav21-dv12	Traj56	Trbv10		Trbj2-5
RP_TCR37	TRA_TRB_1.3_B56_Spleen2	CALSDSNQGGSAKLIF	CASSRTGGAREQYF	Trav6-7-dv9	Traj57	Trbv3		Trbj2-7
RP_TCR35	TRA_TRB_3.1_B56_Spleen3	CAASFYQGGRALIF	CTCSADTGAGNTLYF	Trav7d-4	Traj15	Trbv1	Trbd1	Trbj1-3
RP_TCR32	TRA_TRA_TRB_1.1_B56_Spleen4	CAADNMGYKLTF	CASGGRDRGREQYF	Trav7d-2	Traj9	Trbv13-2	Trbd1	Trbj2-7
RP_TCR33	TRA_TRB_2.4_B56_Spleen5	CILRVPATGGNNKLTF	CASSFNQDTQYF	Trav21-dv12	Traj56	Trbv10		Trbj2-5
RP_TCR41	TRA_TRB_40.1_B56_Spleen6	CASSLPDRGEQYF	CVLVLDTNAYKVIF	Trav6d-5	Traj30	Trbv16	Trbd1	Trbj2-7
RP_TCR42	TRA_TRB_114.1_B56_Spleen7	CALGDWNQGKLIF	CASNRVSAETLYF	Trav6d-6	Traj23	Trbv19	Trbd1	Trbj2-3
RP_TCR38	TRA_TRB_12.1_B56_Spleen8	CAMSRATGGNNKLTF	CASSPGTGNSDYTF	Trav16	Traj56	Trbv16	Trbd1	Trbj1-2
RP_TCR43	TRA_TRB_17.1_B56_Spleen9	CAMREYSGGNYKPTF	CASGGRDRGREQYF	Trav6-3	Traj6	Trbv13-2	Trbd1	Trbj2-7
RP_TCR44	TRA_TRB_7.1_B56_Spleen10	CALSDRGSNAKLTF	CAWTLEDTQYF	Trav6-7-dv9	Traj42	Trbv31		Trbj2-5
RP_TCR45	TRA_TRB_136.1_B56_Spleen11	CAMVGNAGAKLTF	CTCSADGASGNTLYF	Trav13d-1	Traj39	Trbv1	Trbd2	Trbj1-3

Supplementary table 1. TCRs used for hybridomas, isolated from most expanded clones across organs
